# Exploratory Analysis in the Differences in Blood Serum and Seminal Plasma of Adipose-Tissue Related Peptides in Obese and Non-Obese Men and Their Correlations With Semen Parameters

**DOI:** 10.3389/fendo.2021.681939

**Published:** 2021-07-28

**Authors:** Cristina Antinozzi, Marco Lista, Luisa Caponecchia, Pietro Salacone, Carlo Minganti, Francesco A. Battaglia, Luigi Di Luigi, Paolo Sgrò

**Affiliations:** ^1^Unit of Endocrinology, Department of Movement, Human and Health Science, University of Rome “Foro Italico”, Rome, Italy; ^2^Andrology and Pathophysiology of Reproduction Unit, Santa Maria Goretti Hospital, Latina, Italy; ^3^Unit of Sport Medicine, Department of Movement, Human and Health Science, University of Rome “Foro Italico”, Rome, Italy; ^4^Obstetric and Gynecology Unit, Santa Maria Goretti Hospital, Latina, Italy

**Keywords:** male reproduction, adipokines, obesity, sex hormones, incretins

## Abstract

**Objectives:**

Evaluating the relationship between circulating metabolic biomarkers and semen parameters in obese, overweight and normal-weight patients.

**Methods:**

Patients were recruited at the “Andrology and Pathophysiology of Reproduction Unit”, in Santa Maria Goretti Hospital. Divided into three groups were 98 participants (obese, overweight and normal-weight patients) according to BMI and were analyzed for three adipokines and six hormone peptides in blood serum and seminal plasma using Luminex assay. Standard semen analysis was performed for ejaculate volume, sperm concentration, total sperm count, motility, morphology and leukocytes.

**Results:**

In all groups of subjects, we observed a higher concentration of blood serum c-peptide, GIP, PAI-1, leptin, ghrelin and GLP-1 in comparison to seminal plasma; differently, higher levels in seminal plasma were observed for insulin and visfatin. In comparison to the non-obese subjects, obese subjects showed a higher blood serum concentration of c-peptide, GLP-1, GIP and leptin and a higher concentration of seminal plasma of GIP and insulin. Total sperm count, progressive motility, motility, and atypical forms directly correlated with PAI-1 and visfatin, whereas GLP-1 directly correlated only with total progressive motility.

**Conclusion:**

Obese men showed a different pattern of blood serum and seminal plasma adipokines and hormone peptides concentrations in comparison to normal-weight men. Furthermore, these molecules correlated with functional seminal parameters. Our findings support the option to consider these molecules as new biomarkers and pharmacological targets for a new therapeutic approach in male infertility. However, further studies identifying other potential biomarkers of male infertility with important clinical implication and characterizing their mechanisms of action are mandatory.

## Introduction

Obesity has several impacts in society, especially in industrialized countries and is largely associated with other pathological conditions such as atherosclerosis, depression, sleep apnea, type 2 diabetes mellitus (T2DM), cardiovascular and orthopedic disease. A possible relationship between obesity and male infertility has been suggested as well, since the normal function of hypothalamic–pituitary–testicular axis (HPA) depends on the balance of energetic intake and expenditure (1). Studies that investigated the relationship between obesity and male reproductive dysfunction, hypothesized multi-factorial and different pathophysiological causes (2–4). In fact, obesity-induced oxidative stress increases DNA damage in gametic cells (5), interferes with gonadotropin releasing hormone (GnRH) and lutenizing hormone (LH) production, alters spermatogenesis and function of Sertoli cells (6).

Adipokines are cytokines specifically released from adipose tissue to communicate with other organs such as brain, liver, muscle, the immune system and adipose tissue itself (7, 8). The dysregulation of adipokines has been implicated in different metabolic disorders like obesity, T2DM, and cardiovascular disease (7, 8). Recent studies reported the importance of some adipokines and obesity-related hormone peptides in controlling male reproductive system (9, 10), and demonstrated their pivotal role in different molecular mechanisms behind male infertility and sperm functionality (11–13). Indeed, it has shown a high expression of adipokine receptors in seminiferous tubules, specifically in Leydig cells, Sertoli cells and spermatozoa (14), and it has demonstrated a negative correlation between high circulating levels of leptin and ghrelin, and the hormone peptide, ghrelin, with Leydig cells activity and proliferation (15). Thus, interfering with the normal cycle of spermatogenesis and spermatozoa maturation and functionality, these molecules may represent a point of connection that link obesity to male infertility (15, 16). However, the pathophysiological role of these molecules in semen is not clearly elucidated, as well as their possible influence upon sperm functionality. They might be potential biomarkers of male fertility, both in obese or non obese subjects, and this needs to be furtherly investigated. In this preliminary study we assay the concentration of three adipokines—leptin, visfatin, resistin—and six hormone peptides—c-peptide, ghrelin, insulin, GLP-1, PAI-1 and GIP—related to obese and T2DM condition—both in blood serum (BS) and in seminal plasma (SP) of obese, overweight and normal weight subjects. Furthermore, we analyze the correlation between the circulating levels of these molecules with male fertility indices such as serum testosterone, follicle-stimulating hormone (FSH), LH concentration and semen parameters.

## Materials and Methods

### Subjects

We recruited 124 consecutive subjects from the Andrology Unit of Santa Maria Goretti Hospital (Latina, Italy) for analysis of seminal fluid and andrological evaluation, between February 2017 and February 2018. A clinical history was performed to collect personal information including lifestyle factors, sexual and reproductive status and medical history. Eligible patients had not been treated medically or surgically in the previous three months. Exclusion criteria included past medical history of endocrine, oncological or andrological (i.e. varicocele, cryptorchidism) diseases and genital tract infections. FSH, LH and total testosterone were requested by the physician as diagnostic tool of the clinical andrological evaluation while semen examination was performed in our lab. Twenty semen samples containing >1 × 10^6^ leukocytes/ml were excluded as an objective sign of reproductive tract inflammation. Moreover, we excluded from our study five non-obstructive azoospermic samples and one criptozoospermic sample. The remaining 98 participants were divided into three groups according to Body Mass Index (BMI): 34 normal weight (nw) subjects (BMI ≥18.5 ≤24.99 Kg/m^2^); 30 overweight (ow) subjects (BMI ≥25 ≤29.99 Kg/m^2^) and 34 obese (ob) subjects (BMI ≥30 Kg/m^2^). Waist circumference was measured in centimeters at the narrowest point above the hips with the man standing straight. All patients provided informed written consent for personal data treatment in accordance with standard laboratory procedures. Ethical approval was not needed since blood and seminal samples were supplied for clinical assistance and laboratory analysis.

### Semen Analysis

Semen samples were collected by masturbation directly into a sterile plastic container after 3–5 days of sexual abstinence. All samples were left to liquefy at 37°C for 60 min and were then analyzed by the same biologist (LC) by light microscopy according to the World Health Organization criteria (17). The following variables were taken into consideration: ejaculate volume (ml), sperm concentration (n × 10^6^/ml), total sperm count (n × 10^6^/ejaculate), progressive motility (%) and morphology (% abnormal forms). Sperm concentration was examined in a Mackler counting chamber (Sefi-Medical Instrument; Haifa, Israel); sperm motility and morphology were evaluated in duplicates according to standard WHO guidelines. To provide evidence for compliance with WHO recommendations, our andrology laboratory participates in an accreditation program of the Italian Society of Andrology and Sexual Medicine (SIAMS). For each semen sample 1.0 ml of the whole semen was recovered after semen analysis and it was centrifuged at 800*g* for 10 min and then the supernatant was centrifuged again at 3,000*g* for 10 min; the final supernatant was immediately stored at −80°C until adipokines and peptides assays have been carried out.

### Blood Collection

In the same morning of semen analysis, 82 of the 98 subjects have had a blood draw as part of the routine clinical procedure to analyze antisperm antibodies. Venous blood samples were collected *via* venopuncture of superficial vessels by a trained nurse using color-coded Vacutainer tubes. Blood samples were collected after at least 8 h of fasting. To perform adipokine and hormone assay, blood samples were divided according to BMI patients into 26 nw, 30 ow and 26 ob men and were separated by centrifugation at 800*g* for 10 min; 1.0 ml of BS was stored at −80°C until adipokines and peptides assays have been carried out.

### Adipokines and Peptides Measurements

Adipokines and peptides were measured as previously described (18) in all SP and BS samples collected and were measured in triplicate using a magnetic bead-based multiplex assay (Bio-Plex Pro™ Human Diabetes Assay, and Bio-Plex Pro™ Human Hormone Assay, Bio-Rad Laboratories, Inc., USA) according to the manufacturer’s protocol. Data acquisition was performed by a Bio-Plex 200 System™ which uses Luminex fluorescent-bead-based technology (Luminex) to facilitate the analysis of up to 100 different families of color-coded polystyrene beads and allow multiple measurements of the sample ensuing in the effective quantification of adipokines. The Multiplatform luminex assay offers the advantages to use low amount of serum (12.5 to 25 ul of serum per multiplex assay), decreased quality control variability by performing simultaneous assays on a single sample, improved correlated data for multiple analytes, increased sample and finally reduce the manual error during the assay. The working range of the assay is 3,500–280,266 pg/ml with a sensitivity of 0.8 pg/ml and intra-assay CV of 3–4%. Dosage was performed at the Unit of Endocrinology at the University of Rome “Foro Italico”.

### Statistical Analysis

The statistical analysis was performed using GraphPad Prism 5 software (GraphPad Software, Inc., La Jolla, CA, USA) and SPSS 12.0 software package (SPSS for Windows 12.0, SPSS Inc., Chicago, IL, USA). The Kolmogorov–Smirnov test for normal distribution of the data, one-way analysis of variance (ANOVA), and T-test were applied. A *P* value <0.05 was considered significant. Data were expressed as the mean ± standard error (SE). Kruskal–Wallis non-parametric test was used for semen parameters. Data were expressed as median, minimum and maximum. The Pearson correlations among adipokines and serum testosterone data were calculated whereas Spearman correlations among plasma adipokines and semen parameters were calculated.

## Results

**Figure 1:**
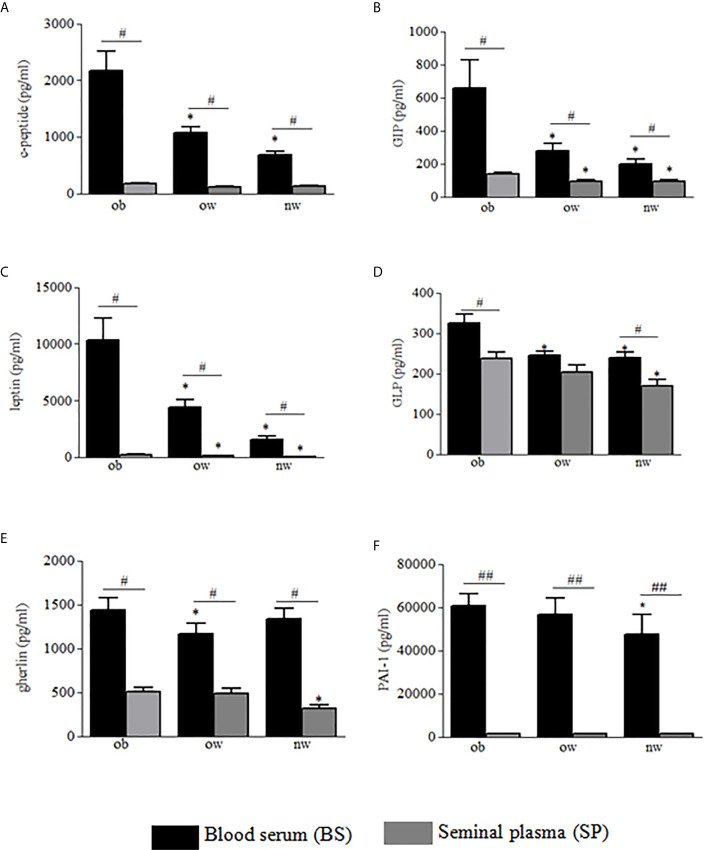
Blood serum (black columns) and seminal plasma (gray columns) of obese, over- and normal weight subjects were analyzed for c-peptide **(A)**, GIP **(B)**, leptin **(C)**, GLP-1 **(D)**, ghrelin **(E)** and PAI-1 **(F)**. Data are presented as the means ± SE. Statistical significance was determined using ANOVA with Bonferroni’s post-hoc test. *P < 0.05 *vs*. obese subjects; *P* < 0.01 *vs.* corresponding adipokine levels between blood serum and seminal plasma. ob, obese; ow, over weight; nw, normal weight. ^##^ *P* < 0.01 vs. corresponding adipokine levels between blood serum and seminal plasma.

**Figure 2:**
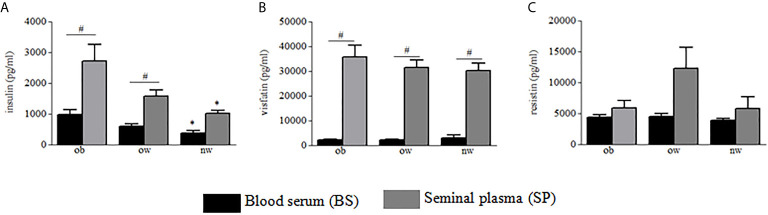
Blood serum (black columns) and seminal plasma (gray columns) of obese, over- and normal weight subjects were analyzed for insulin **(A)**, visfatin **(B)**, and resistin **(C)**. Data are presented as the means ± SE. Statistical significance was determined using ANOVA with Bonferroni’s *post-hoc* test. *P < 0.05 *vs*. obese subjects; ^#^ *P < *0.05 *vs*. corresponding adipokine levels between blood serum and seminal plasma. ob, obese; ow, over-weight; nw, normal weight.

**Figure 3:**
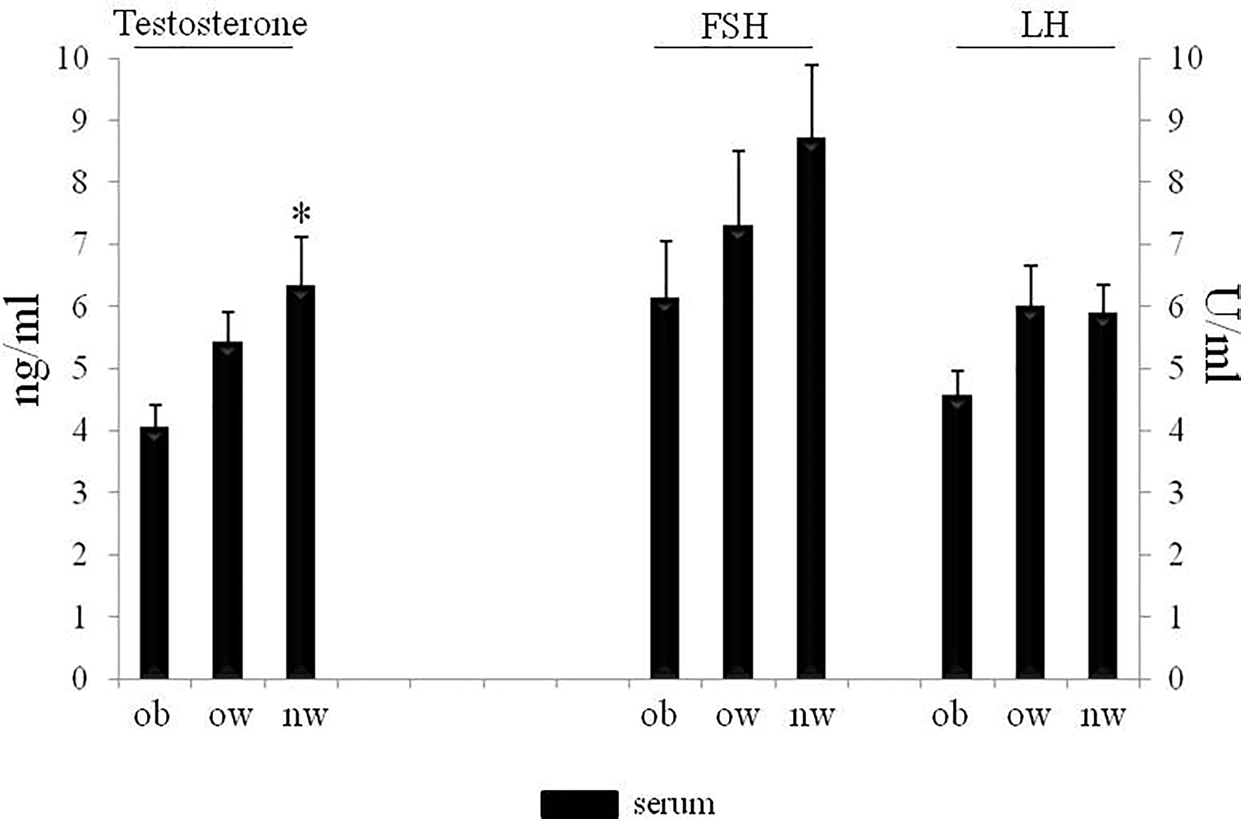
Serum of obese, over and normal weight subjects were analyzed for testosterone, FSH and LH amount. Testosterone is expressed as ng/ml; FSH and LH are expressed as U/ml. Data are presented as the means ± SE. Statistical significance was determined using ANOVA with Bonferroni’s *post-hoc* test. *P < 0.05 *vs*. obese subjects. ob, obese; ow, over-weight; nw, normal weight; FSH, follicle-stimulating hormone; LH, Luteinizing hormone.

Demographic and anthropometric characteristics in obese, overweight and normal weight subjects were reported in **Table 1**. In these subjects, blood serum (BS) and seminal plasma (SP) concentration of specific adipokines and hormone peptides have been investigated and compared. In all three groups of subjects, we observed significant higher levels of BS c-peptide, GIP, leptin, GLP-1, ghrelin and PAI in comparison to SP (**Figures 1A–F**) while a higher statistical significant amount in SP has been observed only for insulin and visfatin (**Figures 2A–C**). Interestingly, by comparing obese and non-obese groups we observed higher levels of BS c-peptide and PAI, BS and SP GIP, GLP-1, leptin, and ghrelin (**Figures 1A–D**), BS and SP insulin (**Figure 2A**) in obese subjects in comparison to the non-obese subjects. By analyzing the levels of the glycoprotein and steroid hormones related to male fertility and reproduction, we observed higher levels of BS testosterone in non-obese subjects in comparison to the obese subjects, while no difference was observed for FSH and LH (**Figure 3**).

**Table 1:** Anthropometric characteristics in obese, over weight, and normal weight subjects.

	Ob		Ow		Nw	
	SP	BS	SP	BS	SP	BS
**Number of subjects**	34	26	30	30	34	26
**Age (y)**	36.5 ± 8.9	37.3 ± 9.3	37.0 ± 5.0	37.0 ± 5.0	36.9 ± 7.5	37.2 ± 7.4
**Height (cm)**	180.6 ± 8.4	181.3 ± 8.1	175.9 ± 7.7	175.9 ± 7.7	178.5 ± 7.3	179.2 ± 7.9
**Weight (kg)**	110.2 ± 14.0	110.4 ± 14.6	84.3 ± 8.1	84.3 ± 8.1	72.9 ± 8.6	73.8 ± 9.3
**BMI (Kg/m^2^)**	33.7 ± 2.9	33.5 ± 2.7	27.2 ± 1.4	27.2 ± 1.4	22.8 ± 1.5	22.9 ± 1.5
**WC (cm)**	103.0 ± 11.4	101.7 ± 11.4	89.8 ± 6.9	89.8 ± 6.9	82.5 ± 5.4	82.8 ± 5.9

Data are expressed as mean ± standard error. ob, obese; ow, over weight; nw, normal weight; SP, seminal plasma; BS, blood serum; BMI, Body Mass Index; WC, Waist circumference.

**Figure 4:**
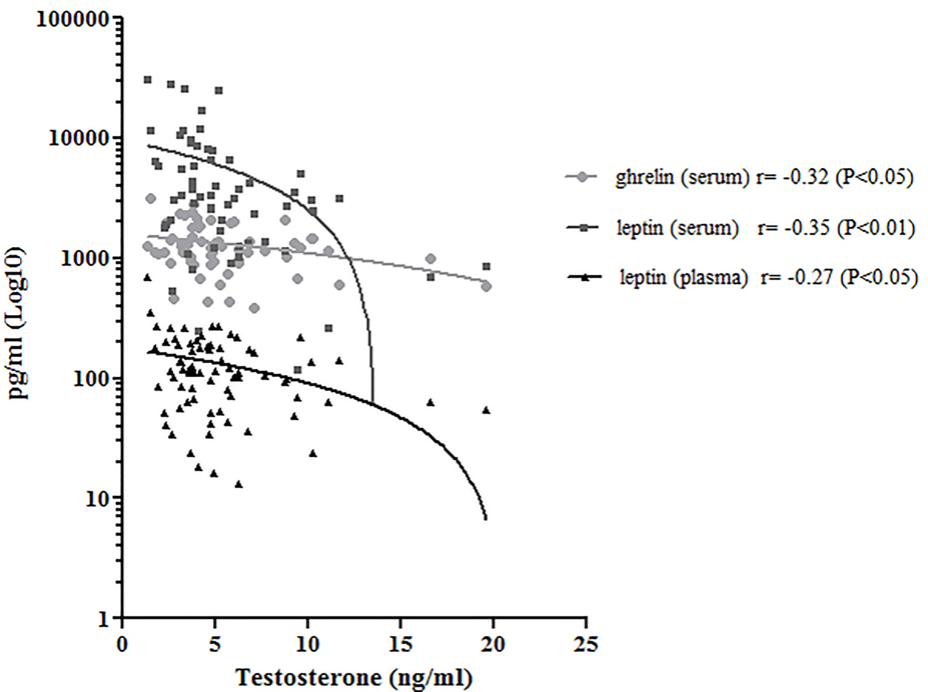
Correlation analysis between serum ghrelin (light gray) and leptin (dark gray), plasma leptin (black) and testosterone. Pearson’s Rho correlation and significance was reported.

Regarding semen analysis, we did not evidence statistical differences in sperm parameters among the three groups (**Table 2**); however, the correlation test between semen and the circulating peptides previously analyzed showed that testosterone correlated negatively with BS ghrelin and with BS and SP leptin (**Figure 4**); furthermore, the number of total spermatozoa and the number of spermatozoa with progressive motility positively correlated with PAI-1(Spearman Rho = 0.342, *P <*0.01 and 0.286, *P <*0.05 respectively) and visfatin (Spearman Rho = 0.414, *P <*0.01 and 0.409, *P <*0.01 respectively), while GLP-1 positively correlates with the number of spermatozoa with progressive motility (Spearman Rho = 0.225, *P <*0.05).

**Table 2:** Semen parameters in obese, over weight, and normal weight subjects.

	Ob	Ow	Nw
**Number of subjects**	34	30	34
**Ejaculate volume (ml)**	3.0 (0.7–6.5)	3.0 (1.5–7.0)	3.5 (2.0–7.0)
**Sperm concentration (n × 10^6^/ml)**	40.0 (0.1–160.0)	30.0 (0.1–160.0)	25.0 (0.1–130.0)
**Total sperm count (n × 10^6^/ejaculate)**	97.5 (0.3–663.0)	84.0 (0.4–595.0)	87.5 (0.55–434.0)
**Progressive motility (%)**	45.0 (0.1–60.0)	40.0 (0.0–55.0)	30.0 (0.0–65.0)
**Atypical forms (%)**	78.0 (68.0–100)	78.0 (68.0–100)	80.0 (72–100)
**Leukocytes (n × 10^6^)**	0.5 (0.2–1.0)	0.5 (0.2–0.9)	0.6 (0.2–1.0)

Data are expressed as median (min and max); ob, obese; ow, over-weight; nw, normal weight.

## Discussion

In this study we demonstrated differences in adipokines and hormone peptides levels in blood sera and seminal plasma of subjects, in association with the BMI. Furthermore, we analyzed and correlated the levels of these circulating molecules with semen parameters and functional characteristics of spermatozoa. Although for a long time adipose tissue was considered an inert organ only aiming to store fat, to date several reports demonstrated that this tissue exerts a pivotal role as endocrine organ, influencing whole body metabolism, nutrient homeostasis, immune response, blood pressure, thyroid and reproductive system function (14, 19–21). The association between fat mass and sperm function is the subject of many studies; however the molecular mechanisms linking obesity and semen quality is not clear yet (22).

The ability of testis cells to produce adipokines has been already demonstrated and it has been showed that these cells can respond to most adipokines and hormone peptides through specific receptors (9, 23). According to these studies, we found that all adipokines measured were detectable in human SP in obese and non-obese subjects, and that for insulin and visfatin molecules SP showed higher levels than in BS. Insulin is important for promoting and regulating growth, differentiation, and metabolism and is mainly produced by β islet cells of pancreas (24). In addition, this hormone plays a central role in the regulation of gonadal function (25). In men affected by insulin-dependent diabetes, sperm have severe structural defects, significantly lower motility (25), and lower ability to penetrate hamster eggs (26). Different studies hypothesize a positive role in improving the fertilization of human spermatozoa by insulin supplementation. Indeed, spermatozoa treated *in vitro* with insulin increase motility, the acrosomal reaction and the production of nitric oxide (27). Sertoli cells have been shown to synthesize and secrete insulin as well (28). By analyzing seminal-to-serum insulin ratio, insulin was found to be highly concentrated in human semen in all groups. However, an interesting datum is the different concentration of c-peptide and insulin in the two fluids. The concentration of insulin in human semen is supported by a limited number of studies, however, and no conceivable explanation for a physiological concentration of insulin in semen is available in literature (29). It is plausible that the amount of SP insulin results from a testis cells production, in particular Sertoli cells (15), and from the ability of this substance to reach seminal plasma through the blood–testicular barrier, seminal vesicles or prostate. The differences between c-peptide and insulin in the two fluids probably come from the much more rapid metabolic clearance of insulin in comparison to c-peptide (30). Consequently, c-peptide has a longer half-life and is present in BS in higher concentrations than insulin.

It is also generally recognized that spermatogenesis is related to the level of testosterone secretion. We observed that serum testosterone negatively correlated with BS and SP leptin and with BS ghrelin. Previous studies, performed in patients affected by azoospermia, demonstrated that these adipokines negatively correlated with testosterone (31). Our study is in agreement with literature, proposing a role of these molecules in metabolic disease affecting male infertility. Leptin receptor is highly expressed in Leydig, Sertoli and presumably in germ cells of different animal models and only in Sertoli cells in human beings (32–34). Fombonne et al. reported that leptin suppressed the division of prepubertal Leydig cells *in vitro* (35). Another study reported that leptin levels were negatively correlated with the levels of inhibin B, the inhibiting hormone of FSH secreted by Sertoli cells (36, 37). It was suggested that leptin may regulate the secretion of inhibin B or may even be related to the regulation of the function of Sertoli cells. This molecule involved in linking energy stores to the reproductive system and presents a positive role in improving the fertilizing ability of human spermatozoa (27). The overexpression of leptin receptors in the testis appears to be related to inhibition of testosterone production (38). It has been demonstrated that high levels of leptin may negatively influence testosterone production inhibiting the conversion of 17-α hydroxyprogesterone in testosterone (39) and that testosterone supplementation has a suppressive effect on leptin production as well (40). We observed higher levels both for SP and BS leptin in the obese subjects and we found that both SP and BS adipokine negatively correlated with testosterone concentration. These results confirm the hypothesis that leptin may be involved in the obesity-related spermatogenic dysfunction. SP ghrelin negatively correlated with serum testosterone as well, and for the first time we found a SP concentration that decreases with body weight. There are no studies that have measured the ghrelin concentration in the SP, although its expression in human testis has been demonstrated (41). It has been speculated that over-expressed ghrelin could lead to inhibition of testosterone production in the human testis (42). Ishikawa et al. did not find any correlation between ghrelin expression in Leydig cells and spermatogenetic activity in normozoospermic patients but showed an inverse correlation between ghrelin expression and testosterone levels (42). Also, in our study the SP ghrelin concentration is higher in obese men who have lower testosterone levels and our observation is in accordance with the concept that this molecule may exert a relevant function in the endocrine network linking the reproductive system control with the regulation of energy balance.

Our results showed a reduction in SP GLP-1 and BS GLP-1 in normal weight patients compared with obese patients; moreover, a positive correlation between seminal GLP-1 and sperm progressive motility has been evidenced. The involvement of GLP-1 in sperm function is not completely understood in literature. Glucagon-like peptide-1 (GLP-1), a strong controller of glucose homeostasis (43), seems also produced in the central nervous system, where it has been implicated in the neuroendocrine control of hypothalamic-pituitary function, food intake and the response to stress (43). GLP-1 stimulates insulin release and inhibits glucagon secretion from the pancreas in response to food ingestion, and its action or effect is impaired in obese subjects (43). Our result shows an increase of GLP-1 in obese subjects. This result could be a compensatory mechanism (44) aiming to maintain GLP-1 action and regulate normal postprandial glycemic response. By analyzing the involvement of GLP-1 in reproduction, it has been observed that male GLP-1 receptor knockout mice exhibited reduced gonadal weights (45). A case report study showed a deleterious effect of liraglutide, an agonist of the GLP-1 receptor, on male reproductive function affecting semen parameters (46) and particularly sperm motility. Finally, a recent study demonstrated the expression of GLP-1 receptor on human spermatozoa, suggesting an important role on sperm motility (47) and our correlation results support the importance of semen GLP-1 for sperm progressive motility. Although a role of GLP-1 in male fertility is still not clear, our study, together with the previous evidences, may be a starting point for further studies evaluating the function of this molecule in male reproduction.

Although its origin still remains uncertain, it has been shown that visfatin is expressed in spermatogonia, spermatocytes, Leydig and Sertoli cells of human testis (48). Only few papers showed an higher visfatin amount in the SP than in BS and a negative correlation between seminal visfatin and ejaculate volume (9). Confirming these studies, our data show a significant higher visfatin concentration in SP compared to BS in all the three groups with similar concentrations. Furthermore, we demonstrated for the first time a positive correlation with some sperm parameters. The high level of visfatin in the SP and its correlation with semen parameters lead as to suppose its possible role in the physiological functions in spermatogenesis and metabolism of the male gametic cell. To support this hypothesis, different *in vitro* studies demonstrated that visfatin increased testosterone production in rat (49), influenced female mouse fertility regulating LH secretion (50) and modulating NAD + levels influencing sperm motility and capacitation (51). Like visfatin, we found a positive correlation between Plasminogen Activator Inhibitor-1 (PAI-1) level and some sperm parameters and, moreover, we found higher levels in BS than in the SP of PAI-1 amount. A previous study analyzed PAI-1 in human semen showing that seminal PAI-1 concentration did not affect human fertility (52). Our results did not only highlight PAI-1 in SP, but also found for the first time a positive correlation between its seminal concentration and the number and the good quality of spermatozoa. Other studies demonstrated that PAI-1 is essential for mammalian spermatogenesis (53). Moreover, it has been demonstrated that PAI-1 was expressed on the external acrosomal membrane of human spermatozoa (54) that could be of some importance for oocyte fertilization.

## Conclusion

In conclusion, although limited for a set of obese- and T2DM-related analytes, our results emphasize the presence in seminal plasma of adipokines and hormone peptides related to diabetes and obese condition, that can, theoretically, influence reproductive function. However, correlating with semen parameters independently from the BMI, some of these molecules in SP probably exert a more direct and specific action in regulating male fertility, compared to the same molecules released in the serum. Moreover, variations in these assayed serum molecule concentrations in obese men can be associated with changes in their seminal concentrations. Importantly, our results show that the influence of these molecules on male infertility can also be independent from body weight since some molecules as GLP-1 and visfatin correlated with sperm parameters independently from metabolic conditions. On the other hand, while obesity influences the serum testosterone levels, it may also affect fertility and sperm quality interfering with the normal function of the negative feedback loop of the HPG axis. One of the limitations of this study is the low number of patients and it is mandatory to validate these data on more representative samples. Furthermore, the analysis of inflammatory markers associated with male sexual function (i.e. as occurs in asymptomatic MAGIs) and oxidative stress (as i.e. interleukin (IL)-6 and IL-8) (55) could be useful to better clarify if in the male obesity, accessory glands secretory function may be also altered. Although some treatments are already available (56), future studies are mandatory to investigate the function of molecules, such as visfatin, PAI-1, GIP and GLP-1, or the function of their receptors in the spermatogenesis and the molecular mechanisms underlying these phenomena.

## Data Availability Statement

The raw data supporting the conclusions of this article will be made available by the authors, without undue reservation.

## Ethics Statement

Ethical approval was not needed since blood and seminal samples were supplied for clinical assistance and laboratory analysis. The patients/participants provided their written informed consent to participate in this study.

## Author Contributions

CA: wrote the manuscript. ML: revised the manuscript. LC: performed experiments. PiS: performed experiments and recruited the patients. CM: performed experiments. FB: revised the manuscript. LL: revised the manuscript. PaS: conceived of the presented idea and wrote the manuscript. LS performed the experiments and wrote and revised the manuscript. All authors contributed to the article and approved the submitted version.

## Conflict of Interest

The authors declare that the research was conducted in the absence of any commercial or financial relationships that could be construed as a potential conflict of interest.

## Publisher’s Note

All claims expressed in this article are solely those of the authors and do not necessarily represent those of their affiliated organizations, or those of the publisher, the editors and the reviewers. Any product that may be evaluated in this article, or claim that may be made by its manufacturer, is not guaranteed or endorsed by the publisher.
